# Efficacy and usability of a novel nebulizer targeting both upper and lower airways

**DOI:** 10.1186/s13052-017-0400-x

**Published:** 2017-09-29

**Authors:** Daniela Posa, Antonio Pizzulli, Petra Wagner, Serena Perna, Stephanie Hofmaier, Paolo Maria Matricardi, Susanne Lau

**Affiliations:** 1 Department of Paediatric Pneumology & Immunology, Charité - Universitätsmedizin, Augustenburger Platz, 1, 13353 Berlin, Germany; 2 Practice for Pediatric Allergy and Pneumology, Berlin, Germany

**Keywords:** Asthma, Children, Nebulizer, Oxymetazoline, Rhinitis, Salbutamol

## Abstract

**Background:**

Upper and lower airways diseases share in part their pathogenic mechanisms and frequently occur simultaneously as “United Airway Disease.” Local treatment with nebulizers delivers anti-symptomatic drugs in either the upper or the lower airways, according to the particle size generated by the nebulizer. To our knowledge, no nebulizer combines both application ways.

The aim of this study is to test the efficacy and usability of a new nebulizer (OMRON A3 complete), generating aerosols with particles diameters of 2-4.5 μm, 4.5-7.5 μm or >7.5 μm, according to the user’s choice.

**Methods:**

Seventy-seven patients between 5 and 17 years of age with a diagnosis of rhinitis or asthma were examined. Oxymetazoline or Salbutamol were prescribed according to best clinical practice guidelines. Both drugs were administered through the OMRON A3 Complete nebulizer, with a particle dimension of >7.5 μm to treat nasal obstruction and 2-4.5 μm for bronchial obstruction. The efficacy of treatment was assessed by total nasal inspiratory airflow and FEV-1, Tiffeneau index (FEV1/FVC) and MMEF 25/75 respectively, 10 min before and after treatment. Symptom improvement and usability were measured by patients’ and doctors’ questionnaires.

**Results:**

Overall, 77 patients seeking care for acute respiratory symptoms were assigned to the upper (*n* = 39) or lower (*n* = 38) airways disease group. For symptoms of the upper airways, 92% (95% CI, 77-97%) of the patients reported subjective improvement, while 87% (95% CI, 73-94%) did so for the lower airways. The average total nasal inspiratory airflow improved significantly (*p* = 0.030) among the patients with upper airways symptoms, from 275 ml/s (95% CI, 207-342 ml/s) to 359 ml/s (95% CI, 300-419 ml/s) after Oxymetazoline administration. All selected lung function parameters (FEV1, Tiffeneau Index and MMEF25-75) significantly improved among the patients with lower airways symptoms after inhalation of Salbutamol (*p* < 0.001). The nebulizer was assessed as “easy to use” by over 95% of participants in both groups.

**Conclusions:**

The OMRON A3 efficiently delivers anti-symptomatic drugs in both upper and lower airways in a user-friendly way. This device may be useful to facilitate adherence to a complete treatment of respiratory symptoms in patients with symptoms of the so-called United Airway Disease.

## Background

Acute and chronic respiratory diseases in childhood are among the first causes of consultation in pediatrics and cause an enormous health and economic burden [1]. Upper (e.g. rhinitis, sinusitis) and lower (e.g. bronchitis, asthma) airways diseases share in part their pathogenic mechanisms and frequently occur simultaneously in the same patient [2–4]. Comorbidity of upper and lower airways has been also defined as “United Airway Disease” (UAD) [5].

To minimize systemic side effects, first-line treatment of airways inflammation is frequently based on local, rather than systemic drugs. Local treatments include corticosteroids and β2-agonists for the lower airways, as well as corticosteroids, nasal anticholinergics and decongestants for the upper airways [2, 3]. Drugs can be administered with metered dose inhalers (MDI), nebulizers, or other devices [6]. The use of MDI is feasible but requires education, adherence and compliance of the patient, while nebulizers are suitable at any age as they do not require special skill in the inhalation technique [7].

Nebulizers are generally divided into two categories, according to the aerosol particle size they produce, i.e. 3-5 μm or 7.5-10 μm to treat either the lower or the upper airways, respectively. Consequently, UAD patients needing local therapy for both upper and lower airways cannot receive their drugs with the same nebulizer.

The primary aim of this study is to test the efficacy of a new nebulizer (OMRON A3 complete) that can be switched to generate aerosols with different particle diameters: 2-4.5 μm, 4.5-7.5 μm or >7.5 μm. The secondary aim of the study is to evaluate whether the use of this new nebulizer is easy and well accepted by patients. To this end, we examined the efficacy and usability of A3C in school children affected by acute exacerbation of asthma or rhinitis. We also assessed the subjective patients’ and doctors’ evaluation of symptoms improvement, acceptability, easiness, and comfort of the use of this new device.

## Methods

### Study population and design

The study population consisted of 77 patients between 5 and 17 years of age seeking care for rhinitis or asthma exacerbations in the Pediatric Outpatient Clinic of the Charité Universitätsmedizin Berlin or in one of its referring practices. The children were eligible for the upper airways study if they had: 1) a diagnosis of rhinitis with no contraindications to the use of local alpha adrenergic drugs; 2) a clear nasal blockage based on clinical assessment; 3) no remarkable anatomical reasons explaining nasal obstruction; 4) no intake of beta-blocker drugs. A 24-hourperiod free of alpha-adrenergic drugs was also required before testing. The children were eligible for the lower airways study if they had: 1) a diagnosis of asthma with no contraindications to the use of beta-2 adrenergic drugs; 2) clinically assessed bronchial obstruction; 3) no remarkable anatomical reasons explaining bronchial obstruction or other severe chronic lung diseases; 4) no intake of beta-blocker drugs. A 12-h period free of long- (LABA) or short-acting β-adrenergic agonists was also required before testing. The study was approved by the local ethic committee. Participants and parents or legal tutors provided informed written consent at the time of enrollment.

### The nebulizer

**Fig. 1:**
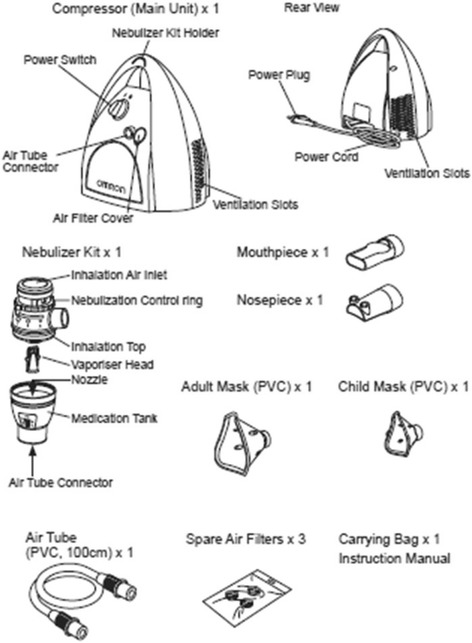
Omron A3C nebulizer Instruction Manual

Drugs were administered through OMRON A3 Complete nebulizer (OMRON, Kyoto, Japan) (Fig. 1). This device produces aerosolized particles with different granulometric characteristics, whose dimensions are changed by switching a “Nebulization Control Ring” (NCR). The NCR in position 1, 2, or 3 produces particles of >7.5 μm, 4.5-7.5 μm or 2-4.5 μm, respectively. In the present study, position 1 was selected to treat nasal obstruction and position 3 was used to treat bronchial obstruction. Further information on the A3C nebulizer can be found at the following web address: https://www.omron-healthcare.com/en/support/manuals/search?utf8=%E2%9C%93&q=NE-C300&commit=%EF%84%B4.

### Drug administration

Children with nasal obstruction were treated with 120 μg Oxymetazoline (6 drops of a solution at 0.05%) + 2.5 ml NaCl 0.9% administered for 5 to 7 min with the A3C nebulizer (NCR in position 1). Children with bronchial obstruction were treated with 1.25 mg Salbutamol (5 drops of a solution at 5 mg/ml) + 2.5 ml NaCl 0.9% administered for 8 to 10 min with the A3C nebulizer (NCR in position 3).

### Endpoints

The efficacy of the treatment with the A3C was measured at the individual level with questionnaires on the patients’ and doctors’ opinion on symptoms improvement. The efficacy of the drug delivery to the upper airways was defined at population level by the increase of the average total nasal inspiratory airflow (V’na) after administration of Oxymetazoline with the A3C (see below for details). V’na was measured according to standard procedures [8] with Rhinomanometry (ZAN 100, N-spire Health, Germany) 10 min before and after the treatment with Oxymetazoline. Participants were asked to blow their nose before starting the assessment and to breath normally with the mouth closed during the measurement. Subjects were invited to introduce in the nostrils the two plastic olives attached to flow and pressure sensors of the Rhinomanometry. Each nostril was evaluated separately [9].

The efficacy of drug delivery to the lower airways was defined at population level by the increase of FEV-1, Tiffeneau Index (FEV1/FVC) and Maximal Midexpiratory Flow (MMEF 25/75) after administration of Salbutamol with the A3C. These parameters were measured with a Spirometer (ZAN 100, N-spire Health, Germany) 15 min before and after the treatment with Salbutamol. The doctors’ and patients’ opinions on the acceptability, easiness, and comfort of the use of the A3C were assessed through questionnaires. Patients were also asked to estimate their satisfaction of the use of the nebulizer through a Visual Analogue Scale (VAS).

### Statistical analysis

Data were summarized as numbers (n) and frequencies (%) if they were categorical and as mean and standard deviation (SD) if quantitative. To assess the normal distribution of quantitative data, the Shapiro–Wilk test was applied. A paired-samples t-test, when conditions were respected or Wilcoxon signed-rank test were performed to show differences in time within each group of patients. A *p*-value <0.05 was considered statistically significant. Statistical analyses were performed with R statistical software (R Core Team, 2014), version 3.2.3.

## Results

### Characteristics of the study population

Overall 87 patients seeking care for acute respiratory symptoms were examined, of which 77 met the inclusion criteria to be assigned to the upper (*n* = 39) or to the lower (*n* = 38) airways disease group (Table 1). Allergic rhinitis (33/39; 85%) and allergic asthma (31/38; 82%) were the most frequently diagnosed diseases in the group with upper and lower airways disease, respectively. An allergic reaction or a viral infection were the most common triggers of the acute exacerbation to be treated. The severity of symptoms to be treated was classified as “moderate” by the patients in 79% and 42% of those having upper or lower airways symptoms, respectively. Symptoms were more frequently short lived (1-3 days) in the group with lower airways disease than in that with upper airways disease (55% vs 10%, *p* < 0.001), but the latter suffered more frequently from sleep impairment (46% vs 34%, *p* = 0.285) (Table 1).

**Table 1:** Characteristics of the study population and of the current disease exacerbation

	rhinitis		asthma	
	*n* = 39		*n* = 38	
Male gender, n (%)	25	64	27	71
Age (yrs), mean (SD)	9	3.0	10	3.0
Height (cm), mean (SD)	139	17	143	18
Weight (kg), mean (SD)	37	18	39	16
Atopic diseases, n (%)				
Allergic rhinitis	33	85	21	55
Allergic asthma	24	62	31	82
Etiology, n (%)^a^				
Viral	24	62	11	29
Bacterial	1	3	1	3
Allergic	27	69	26	68
Other	4	10	8	21
Severity, n (%)				
low	1	3	13	34
medium	31	79	16	42
high	4	10	7	18
very high	3	8	2	5
Duration, n (%)				
1-3 days	4	10	21	55
4-7 days	12	31	6	16
7-15 days	11	28	8	21
> 15 days	12	31	3	8
Impaiment of sleeping, n (%)	18	46	13	34
Impairment of eating, n (%)	0	0	3	8

Data were summarized as numbers (n) and frequencies (%) if they were categorical and or mean and standard deviation (SD) if quantitative

^*a*^More than one etiology is possible in the same subject

### Efficacy

**Fig. 2:**
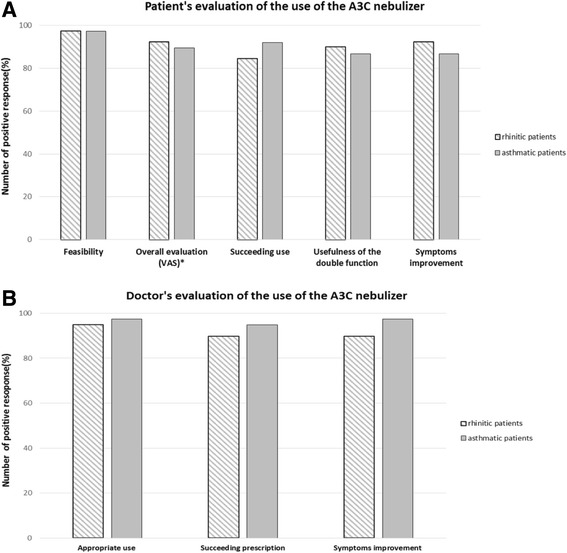
Subjective evaluation (**a**) Subjective patient’s evaluation of the use of the A3C nebulizer in 39 rhinitic patients and in 38 asthmatic patients. A response between 1 and 4 in a Visual Analogue Scale from 1 to 10 was considered as positive. **b** Doctor’s evaluation of the use of the A3C nebulizer in 39 rhinitic patients and in 38 asthmatic patients

The percentage of patients reporting a subjective feeling of clear symptoms improvement was 92% (95% CI, 77-97%) and 87% (95% CI, 73-94%) in the upper and lower airways disease groups, respectively (Fig. 2). Doctors judged a clear improvement of symptoms in 90% (95% CI, 76-96%) and 97% (95% CI 87-100%) of the patients with upper and lower airways disease, respectively (Fig. 2).

**Fig. 3:**
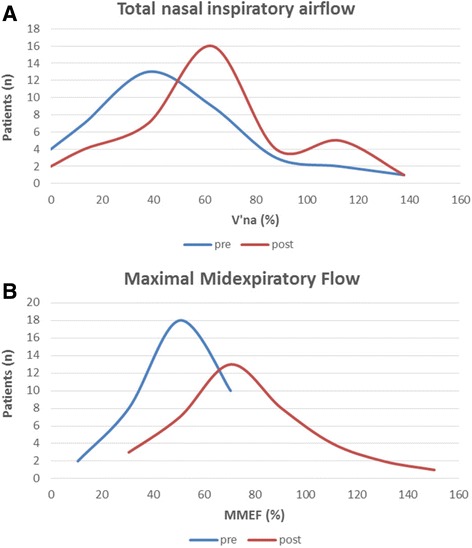
Objective evaluation of (**a**) quantitative improvement of the Total Nasal Inspiratory Airflow at population level and (**b**) quantitative improvement of the Maximal Midexpiratory Flow at population level. **a** Smoothed frequency distribution of V’na values (expressed as percentage (%) of the expected value) measured before (pre) and after (post) nebulization. **b** Smoothed frequency distribution of MMEF 25/75 values (considered as percentage (%) of the expected values) measured before (pre) and after (post) nebulization

The average total nasal inspiratory airflow significantly improved (*p* = 0.030) among the patients with upper airways symptoms, from 275 ml/s (95% CI, 207-342 ml/s) to 359 ml/s (95% CI, 300-419 ml/s) after Oxymetazoline administration with the A3C nebulizer (Table 2). The frequency distributions of V’na values measured before and after nebulization were notably different. Particularly, V’na values evaluated after nebulization were distributed towards higher values, with a peak in interval between 51 and 75 V’na (%). Moreover, the mean differences resulted statistically significant (44% vs 61%, *p* = 0.010) (Fig. 3a).

**Table 2:** Quantitative improvement of respiratory parameters in 39 rhinitic patients and in 38 asthmatic patient after drug administration with A3C nebulizer

	pre		post		*p*-value^a^
	mean	SD	mean	SD	
Rhinitic patients					
Total nasal inspiratory airflow (ml/s)	275	216	359	188	0.030
Asthmatic patients					
FEV 1 (l)	1.59	0.63	1.90	0.80	<0.001
Tiffeneau index (%)	78	11.0	87	10.1	<0.001
MMEF 25-75 (l/s)	1.32	0.50	2.01	0.85	<0.001

Quantitative data were summarized as mean and standard deviation (SD)

^a^A paired t-test, when condition were respected (Shapiro-Wilk test was used to assess normality of data), or Wilcoxon signed-rank test was performed to show differences in time within each group

All the selected lung function parameters (FEV1, Tiffeneau Index and MMEF25-75) significantly improved among the patients with lower airways symptoms after inhalation of Salbutamol (Table 2). Similarly, MMEF 25/75 values measured after nebulization revealed a tendency towards higher values, with a peak in the interval between 61 and 80 MMEF 25/75 (%).The mean pre-post nebulization differences were statistically significant (51% vs 77%, *p* < 0.001) (Fig. 3b).

### Usability

Only 1/39 patients with upper airway disease (3%) and 2/38 patients with lower airway disease (5%) reported a previous use of the A3C nebulizer. The nebulizer was assessed as “easy to use” by 97% of the participants in both groups (Fig. 2a). 85% and over 92% of the participants with disease of the upper or lower airways, respectively, expressed the wish to use the nebulizer again. More than 85% of the patients affected by UAD considered the possibility of using one single nebulizer to treat their symptoms of both upper and lower airways as useful. Overall, only seven patients evaluated the use of the nebulizer as “uneasy” (Fig. 2a). Doctors did not indicate any problem in the patients’ use of the device for both groups and would have prescribed the use of the nebulizer for over 90% of their patients again (Fig. 2b).

## Discussion

### What we found

We tested the efficacy and usability of a novel nebulizer (A3 complete, OMRON, Kyoto, Japan) in two groups of children with either upper or lower airways disease. In both groups the nebulizer was considered as “easy to use” by the patients. The successful delivery of the anti-symptomatic drug (Oxymetazoline or Salbutamol) was assessed at individual level through the subjective opinion of the patients and their doctor. At population level, the success of drug delivery in the target tissue was measured by the improvement of objective upper or lower airways parameters, i.e. total inspiratory nasal airflow and lung function parameters. (FEV1, Tiffeneau Index, MMEF 25/75) To our knowledge, this is the first example of a nebulizer being able to effectively deliver drugs to both the upper and lower airways; actually, no nebulizer with this property has been mentioned in a recent, comprehensive review on nebulizers [10].

### Drug deposition in upper/lower airways

The efficacy of drug delivery in the lower airways was clearly detected at population level in terms of variation of FEV-1. This increase was remarkably evident when the Maximal Midexpiratory Flow (MMEF25-75) was taken into account. This suggests a successful penetration and deposition of the drug (Salbutamol) in the targeted segment of the airways, i.e. the medium-size and smaller bronchi. Accordingly, this improvement was also accompanied by a clear improvement of the Tiffeneau Index. Most of the patients subjectively reported improvement of their bronchial symptoms, assessed both through specific questioning (87%) and a visual analogue scale (89%). The efficacy of drug delivery in the upper airways was detected at population level by measuring the total nasal inspiratory flow. This objective improvement suggests the effective penetration and deposition of the drug (Oxymetazoline) in the upper airways. Accordingly, most patients subjectively reported improvement of their nasal symptoms.

### Usability of the device for patients with UAD

The improvement of subjective and objective symptoms reflects why patients and doctors consider the use of the nebulizer successful. Interestingly, the patients with symptoms in both upper and lower airways expressed appreciation for the possibility of treating both affected sites with a unique nebulizer. Additionally, the instrument seemed to be user-friendly for most of the participating patients. In certain patients (e.g. pre-school children, patients with difficult coordination or low attention) the use of a single and easier device, which is able to treat both the upper and lower airways, may be preferable and facilitate adherence to a complete treatment of the so-called “United Airways Disease” [11, 12].

### Choosing the appropriate device for each patient

Although the use of the A3C has been shown to be beneficial for patients with rhinitis and asthma, one should consider that nebulizers generally are not the most frequently used drug delivery devices in the treatment of both pathologies [13]. In particular, the European and German national guidelines for the treatment of rhinitis and asthma suggest as first choice the use of nasal drops/sprays and metered dose inhalers with aero-chamber to deliver anti-symptomatic drugs or corticosteroids in the upper and lower airways, respectively [14, 15].

One of the reasons for this recommendation is that most nebulizers are relatively big, not easily carried instruments, requiring electric power [16]. This impedes an outdoor use, e.g. during sports or at school [17], which is an essential limiting factor with regard to the treatment of acute disease exacerbations, especially in asthma. Moreover, some nebulizers have a relatively high degree of variability in their efficiency of drug delivery over time [18].

### Study limitations

We have to acknowledge some limitations of our study. First, we did not compare the efficacy of A3C with that of other nebulizers. Nevertheless, the very high efficacy measured suggests non-inferiority to other nebulizers already available on the European market in delivering drugs to either upper or lower airways. Second, we measured the impact of therapy only in not-hospitalized patients affected by milder forms of acute exacerbations of respiratory diseases. We stress that our conclusions cannot be automatically extrapolated, without further studies, to more severe conditions, such as severe asthma, cystic fibrosis or to use in unconscious patients. Third, the subjective opinion of patients and doctors and the objective measures of lung or nasal function were based on one episodic treatment only, without further follow-up assessments. A study evaluating the efficacy of the instrument on patients monitored long-term would have been more informative.

## Conclusions

In conclusion, this study has shown that the A3C nebulizer efficiently delivers anti-symptomatic drugs in both the upper and lower airways in a user-friendly way. This device may be useful to facilitate adherence to a complete treatment of respiratory symptoms in patients with symptoms of both upper and lower airways symptoms, i.e. the so-called United Airways Disease.

## Abbreviations

A3C: OMRON A3 complete nebulizer
CI: Confidence interval
FEV1: Forced expiratory volume in 1 s
FVC: Forced vital capacity
MDI: Metered dose inhalers
MMEF 25/75: Maximal midexpiratory flow
NCR: Nebulization control ring
SD: Standard deviation
UAD: United airway disease
V′na: Total nasal inspiratory airflow
VAS: Visual analogue scale
